# New Green Biorefinery Strategies to Valorize Bioactive Fractions from *Palmaria palmata*

**DOI:** 10.3390/md22100467

**Published:** 2024-10-11

**Authors:** Melis Cokdinleyen, Gloria Domínguez-Rodríguez, Huseyin Kara, Elena Ibáñez, Alejandro Cifuentes

**Affiliations:** 1 Laboratory of Foodomics, Institute of Food Science Research (CIAL, CSIC), Nicolás Cabrera 9, 28049 Madrid, Spain; melis.cokdinleyen@cial.uam-csic.es (M.C.); elena.ibanez@csic.es (E.I.); a.cifuentes@csic.es (A.C.); 2 Faculty of Sciences, Department of Chemistry, Selçuk University, Arciçh, Ismetpasa Cad, Selçuklu, Konya 42250, Turkey; hkara@selcuk.edu.tr; 3 Departamento de Química Analítica, Química Física e Ingeniería Química, Facultad de Ciencias, Universidad de Alcalá, Ctra. Madrid-Barcelona Km. 33.600, Alcalá de Henares, 28871 Madrid, Spain

**Keywords:** biorefinery, phycobiliproteins, sulfated polysaccharides, proteins, phenolic compounds, *Palmaria palmata*, natural deep eutectic solvents, ultrasound-assisted extraction, pressurized liquid extraction

## Abstract

A biorefinery process was developed to isolate phycobiliproteins, sulfated polysaccharides, and phenolic compounds from *Palmaria palmata*. The extraction process was carried out in three stages using ultrasound-assisted extraction (UAE) and pressurized liquid extraction (PLE) integrated with different natural deep eutectic solvents (NaDESs). In general, PLE provided higher phycobiliprotein contents than UAE in the first step of the process. In fact, the hydrolysis product of the PLE-NaDES extracts achieved a higher antioxidant capacity than that of the UAE-NaDES extracts. Particularly, glycerol:glucose (2:1) with 50% water in combination with PLE was the most suitable NaDES to recover the highest phycobiliprotein, protein, and sulfated polysaccharide contents from *Palmaria palmata* in the first and second steps of the biorefinery process. Finally, a PLE-NaDES using choline chloride:glycerol (1:2) with 60% water as the NaDES was employed for the recovery of antioxidant and neuroprotective phenolic compounds from the residue of the second step, obtaining a higher total phenolic content than employing PLE with ethanol/water (70:30, *v*/*v*) as the extraction solvent. Moreover, a forced stability study revealed that the NaDESs provided a protective effect compared to the water extracts against the degradation of phycobiliproteins, preserving their color over time. This study contributes to the recovery of high-value components from an undervalued biomarine source through a sustainable biorefinery process.

## 1. Introduction

Currently, the Food and Drug Administration (FDA) as well as the European Food Safety Authority (EFSA) are introducing regulations to limit the use of synthetic colors in foods due to health concerns, such as quinoline yellow colorant, which induces toxicity by protein aggregation [1]. Red macroalgae have recently attracted significant attention in the exploration of natural colorants with bioactive properties for their inclusion as innovative additives for pharmaceutical and food products [2,3].

*P. palmata* is a red macroalga that grows from the middle of the intertidal zone (the region between high and low tide), in both sheltered and exposed coastal areas [4]. The current availability of *P. palmata* depends on harvesting wild populations, but it is difficult to meet growing demand [5]. Currently, *P. palmata* aquaculture is largely small-scale, lacks significant mechanization, and is primarily carried out during spring and summer, when the plants are more easily harvested by hand [6]. Thus, different researchers have focused their efforts on the development of the vegetative propagation and cultivation processes of *P. palmata* to enhance its use in commercial-scale operations [6,7]. However, the current cultivation of this alga has been scarcely exploited as a source of natural pigments and phenolic compounds despite its interesting content [2,8]. Particularly, the major constituents of *P. palmata* are pigment proteins called phycobiliproteins.

Phycobiliproteins are light-harvesting pigment proteins with high levels of solubility that are found intracellularly in red algae [9]. These proteins consist of apoproteins and chromophores linked by thioether bonds, which exhibit different color properties due to the presence of four different chromophores in their structure. According to their structure and absorption spectra, phycobiliproteins are divided into phycoerythrin (490–570 nm), phycocyanin (610–625 nm), phycoerythrocyanin (560–600 nm), and allophycocyanin (650–660 nm) [10,11]. Frequently, they are obtained from red algae such as *P. palmata* to be included in food as colorants and functional ingredients [9,11]. In fact, phycobiliprotein hydrolysates from *P. palmata* have shown interesting anti-inflammatory and antihypertensive capacities [12,13,14]. However, the bioactivity of these compounds obtained from *P. palmata* has scarcely been studied.

These compounds are usually recovered from algae employing conventional extraction, such as maceration, with water as the solvent; however, this approach has several limitations [10]. Phycobiliproteins are found intracellularly, and their extraction implies the rupture of the rigid cell wall of red macroalgae to break the polysaccharide barrier, releasing phycobiliproteins as well as valuable proteins and sulfated polysaccharides to the extraction solvents [15,16,17]. In this sense, conventional extractions with water are often inefficient, and more aggressive extraction treatments are necessary to break down the cell wall. For that, freeze grinding or maceration with liquid nitrogen, freezing, and thawing processes are employed to break down the algae cell wall, improving the recovery of phycobiliproteins [10,17,18]. Nevertheless, these treatments require the use of costly specific instruments as well as large extraction times, implying that phycobiliproteins are exposed to light and oxygen for long times, increasing their susceptibility to degradation [10,19]. For this reason, advanced extraction techniques such as enzymatic-assisted extraction (EAE), ultrasound-assisted extraction (UAE), or pressurized liquid extraction (PLE) have been proposed by several authors as more intense, selective extraction techniques that require lower extraction times and temperatures compared with conventional extractions [18,20,21,22]. Particularly, considering the composition of the cell wall of the algae, specific enzymes can be selected to break it down and release target compounds by EAE; however, this type of extraction technique requires the precise control of pH, temperature, and substrate concentration. Moreover, the use of specific enzymes makes the process more expensive [2]. On the other hand, the use of ultrasonic waves in UAE and the high pressure employed in PLE have demonstrated the breakdown of the biomass of different algae, favoring the release of phycobiliproteins [17,23,24]. In fact, Gallego et al. [24] developed a biorefinery process to obtain phycobiliproteins, proteins, and sulfated polysaccharides from *Porphyridium cruentum* biomass by PLE using water as an extraction solvent. In addition, the biorefinery process developed by Gallego et al. [24] revealed the importance of the use of a biorefinery process to extract and fractionate valuable compounds from algae for their integration into sustainable biomass production procedures.

Advanced extraction techniques such as UAE and PLE have been employed with water as the extraction solvent for the recovery of phycobiliproteins, proteins, and sulfated polysaccharides [23,24]. Phycobiliproteins are hydrosoluble molecules, and this makes water an ideal solvent for releasing these proteins from the algae cells. Nevertheless, solvent selection is not only a crucial parameter for achieving a high extraction efficiency but it also impacts the stability of target compounds after extraction. In this sense, water has not demonstrated a protective effect on the degradation of bioactive compounds from different matrices [25], and considering that the phycobiliproteins’ color is unstable to heat and light, the selection of solvents with preservative effects could be an interesting approach [19]. In particular, sustainable, biodegradable, non-toxic, and economic solvents with preservative effects on the degradation of bioactive compounds have emerged in the past few years: natural deep eutectic solvents (NaDESs) [25,26].

NaDESs are mixtures of natural components, such as sugars, amino acids, or organic acids, among others, which have demonstrated a significant potential for the extraction process due to the strong hydrogen bonds formed between the solvent components and the extracted compounds [26]. These interactions provide higher extraction yields of bioactive compounds compared to the use of conventional solvents such as water as well as a protective effect against their degradation [27]. Moreover, NaDESs possess a higher viscosity than water; this reduces the mobility of target compounds within the solution, limiting their exposure to oxygen and consequently the oxidative reactions [28]. Even though these solvents have been widely employed for the recovery of bioactive compounds (such as phenolics) from several food matrices using maceration or combined with advanced extraction techniques, such as UAE and PLE [29], studies about the recovery of bioactive compounds from algae are very limited [30,31]. In fact, there are no studies about the recovery of phycobiliproteins, proteins, sulfated polysaccharides, and phenolic compounds from *P. palmata* by NaDESs combined with advanced extraction techniques.

Thus, this study proposes, for the first time, the development of a biorefinery process for the recovery of phycobiliproteins, proteins, sulfated polysaccharides, and phenolic compounds from *P. palmata* based on the combination of UAE and PLE with NaDESs. In the first and second steps of the biorefinery process, different NaDESs were tested to maximize the extraction of phycobiliproteins, proteins, and sulfated polysaccharides with antioxidant capacity in combination with UAE and PLE. Finally, in the third step, phenolic compounds were obtained employing PLE-NaDESs with choline chloride:glycerol (1:2) as the NaDES. The extraction efficiency of the NaDES was evaluated by comparison with water extracts, together with its preservative effect on the degradation of phycobiliproteins during a forced stability study.

## 2. Results

**Figure 1:**
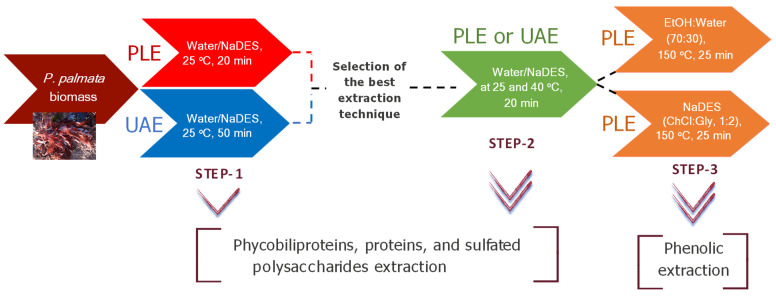
Workflow followed for the valorization of *Palmaria palmata* biomass through a biorefinery process using UAE and PLE combined with NaDES.

To develop a biorefinery approach for extracting valuable compounds from *P. palmata* biomass, a sequential downstream process was established according to Figure 1. For that, the characteristics of the target compounds, the use of environmentally friendly solvents, and the need for a rapid and efficient extraction method were investigated. Based on these considerations, experiments were conducted to identify the optimal advanced extraction technique for maximizing thermolabile phycobiliproteins’ recovery (step 1) followed by maximizing the recovery of residual non-thermolabile phycobiliproteins along with proteins and sulfated polysaccharides (step 2), and finally, phenolic compounds (step 3).

### 2.1. Optimization of Biorefinery Process

#### 2.1.1. UAE-NaDES and PLE-NaDES Extraction of Bioactive Phycobiliproteins, Proteins, and Sulfated Polysaccharides from *P. palmata* (Step 1)

One of the most important physicochemical parameters that determine the extraction efficiency is the solvent viscosity, which is widely modified by the addition of water. For this reason, different NaDESs were synthesized with two different water percentages (25% and 50%), considering that hydrogen bonds of NaDESs are able to stay stable up to this percentage [32].

NaDES viscosity was measured at 25 °C and 40 °C (see Table S1), observing that when the temperature and the percentage of water in the NaDES increased, the NaDES viscosity decreased. However, different authors have reported that the optimal extraction conditions for the recovery of thermolabile phycobiliproteins such as phycoerythrin are 25 °C using water for the extraction [24,33]. For this reason, step 1 of the biorefinery process was focused on the extraction of phycobiliproteins at low temperatures, avoiding their degradation.

UAE-NaDES and PLE-NaDES extractions were compared in terms of phycobiliprotein contents; however, due to the chemical composition of *P. palmata* [5,34], other compounds such as proteins and sulfated polysaccharides can be co-extracted. Thus, these compounds were also analyzed.

In particular, Gly:Glu:Bet and Gly:Glu:Pro NaDESs with 25% water could not be implemented in step 1 of the biorefinery process using PLE by obstruction problems.

As can be observed in Table 1, the use of higher percentages of water in NaDESs increased the recovery of phycobiliproteins for both extraction techniques, except for the recovery of APC by UAE, in which 25% water in the NaDES allowed us to obtain higher APC contents than using 50% water. Particularly, the PLE-NaDES using Gly:Glu with 50% water as the NaDES was the best extraction technique for the recovery of phycobiliproteins from *P. palmata* compared to the UAE-NaDESs. Moreover, the efficiency of the NaDES as a solvent extraction to obtain phycobiliproteins was confirmed by comparisons with UAE-water and PLE-water extractions. As can be seen in Table 1, the use of water as an extraction solvent provided lower phycobiliprotein values than using the Gly:Glu NaDES. Nevertheless, the UAE-NaDES achieved the highest protein and sulfated polysaccharide contents compared to the PLE extractions, except for the PLE-water extraction.

In agreement with the phycobiliprotein extraction, the NaDES composed of Gly:Glu with 50% water allowed us to obtain the highest total protein and sulfated polysaccharide contents by UAE, but without statistically significant differences with the UAE-water extraction in terms of protein content. Lower protein and sulfated polysaccharide contents were achieved by PLE than by UAE, with the PLE-water extracts having higher contents than the PLE-NaDES extracts. Probably, an increase in the extraction temperature could improve the recovery of proteins and sulfated polysaccharides by PLE.

On the other hand, considering that the NaDES with 50% water provided extracts containing high phycobiliprotein, protein, and sulfated polysaccharide contents, these extracts were hydrolyzed with trypsin in order to investigate their antioxidant capacity. To determine the optimum hydrolysis time with trypsin, the antioxidant capacity of the hydrolysates was evaluated after hydrolysis at different incubation times (1, 3, 5, and 6 h) (Table S2). As a result, a hydrolysis time of 1 h was established since there was not a significant increase in the antioxidant capacity after this incubation time. Therefore, the antioxidant capacity of all extracts was evaluated by TEAC and ORAC assays after 1 h of hydrolysis with trypsin.

Table 2 shows that the antioxidant capacity of the extracts increased after the hydrolysis treatments. Particularly, the PLE-NaDES hydrolysates exhibited higher antioxidant capacity than the UAE-NaDES hydrolysates, highlighting those obtained with the Gly:Glu:Bet (4:1:1) NaDES with 50% water. However, the PLE-water hydrolysates presented the highest antioxidant capacity determined by the ORAC assay compared to the PLE-NaDES hydrolysates.

#### 2.1.2. PLE-NaDES Extraction of Phycobiliproteins, Proteins, and Sulfated Polysaccharides (Step 2)

Considering the potential protein and sulfated polysaccharide contents of *P. palmata*, their extraction along with the remaining non-thermolabile phycobiliproteins from the residual biomass of step 1 was considered to obtain high value-added products. For that, the residual biomass from the application of step 1 (the PLE-NaDES composed of Gly:Glu with 50% water) was studied for step 2.

Step 2 was applied considering six different NaDESs at two different PLE extraction temperatures (25 °C and 40 °C), but excluding the NaDESs composed of Gly:Glu:Bet and Gly:Glu:Pro with 25% water, as previously mentioned. The PLE-NaDES extracts were compared with the PLE-water extracts obtained under the same extraction conditions.

Table 3 shows that the extracts exhibited higher phycobiliprotein contents than in step 1 (see Table 1). These results indicated that phycobiliproteins remain retained in the residual biomass of step 1, and additional steps are required to completely exploit the biomass. As can be seen in Table 3, similar phycobiliprotein results were obtained using PLE at 25 °C and 40 °C with NaDESs and water solvents, except when Gly:Glu with 50% water was used, which provided the extracts with the highest phycobiliprotein contents when an extraction temperature of 40 °C was applied. The Gly:Glu NaDES with 50% water was the only one that improved the extraction of phycobiliproteins, showing a protective effect against the degradation of these compounds when an extraction temperature of 40 °C was used compared with the rest of the NaDESs. In fact, the extracts obtained at 40 °C with the other NaDESs and with water showed similar values or even lower values than those obtained at 25 °C. In addition, even though the PLE-water extracts presented higher total protein and sulfated polysaccharide contents than the PLE-NaDES extracts, the PLE-water extracts showed lower phycobiliprotein contents.

Considering these results, the PLE-NaDES extraction using Gly:Glu with 50% water at 40 °C was selected as the best to obtain the richest extracts in phycobiliproteins with significant amounts of proteins and sulfated polysaccharides.

#### 2.1.3. PLE-NaDES Extraction of Bioactive Phenolic Compounds (Step 3)

The third and last step of the biorefinery process focused on the recovery of bioactive phenolic compounds from the residual biomass of step 2. For that, the PLE-NaDES extraction with ChCl:Gly (1:2) with 60% water as the extraction solvent at 150 °C for 25 min was applied for the recovery of phenolic compounds according to Domínguez-Rodríguez et al.’s [35] method for the recovery of antioxidant and anticholinergic phenolic compounds from food matrices. In addition, the same PLE extraction conditions were employed, but ethanol/water (70:30, *v*/*v*) was used as the extraction solvent instead of the NaDES in order to compare both extracts in terms of their total phenolic contents and their antioxidant and anticholinergic capacities.

**Figure 2:**
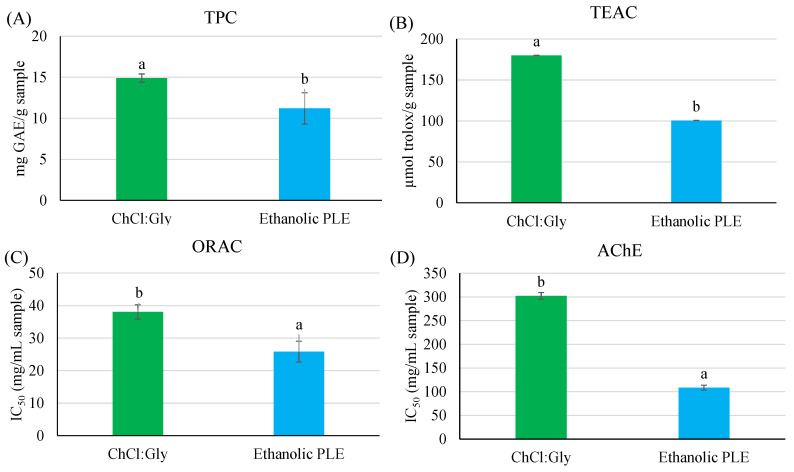
(**A**) Total phenolic content (TPC) determined by Folin–Ciocalteu method, antioxidant capacity measured by (**B**) TEAC and (**C**) ORAC assays, and (**D**) anticholinergic capacity evaluated by the inhibition of AchE enzyme from phenolic extracts obtained by PLE-NaDES with ChCl:Gly (1:2) with 60% water and PLE with ethanol/water (70:30, *v*/*v*). ^a,b^ Letters indicate statistically significant differences between extraction solvents (*p* ≤ 0.05).

As can be observed in Figure 2, the PLE-NaDES extraction provided higher TPC values (Figure 2A) and a higher antioxidant capacity than the ethanolic PLE extraction, determined by the TEAC assay (Figure 2B). However, the ethanolic extract presented a higher antioxidant capacity evaluated by the ORAC assay (see Figure 2C) as well as a higher anticholinergic capacity (see Figure 2D). This means that the PLE-NaDES extraction with ChCl:Gly (1:2) with 60% water or the ethanolic PLE extraction can be used depending on the final application added to the extract.

### 2.2. Stability Study of PLE-NaDES and PLE-Water Phycobiliprotein Extracts

The selection of an appropriate solvent for the extraction of bioactive compounds significantly impacts its stability post-extraction. Numerous studies have demonstrated that NaDESs are particularly effective in preventing the degradation of bioactive compounds, such as phenolic compounds, under forced storage conditions [36,37]. However, to our knowledge, there are no studies about the stability of phycobiliproteins achieved using NaDESs as the extraction solvent from *P. palmata* algae. Thus, a stability study of the optimal PLE-NaDES extraction (using Gly:Glu (2:1) NaDES) and the PLE-water extracts obtained in step 1 was performed by monitoring the phycobiliprotein contents (B-PE, R-PC, and APC contents), the antioxidant capacity of their hydrolysates (determined by TEAC and ORAC assays), and the color change (measured by CIELAB coordinates) for 30 days. During these 30 days, the extracts were submitted to 40 °C, equivalent to 6 months at room temperature.

**Figure 3:**
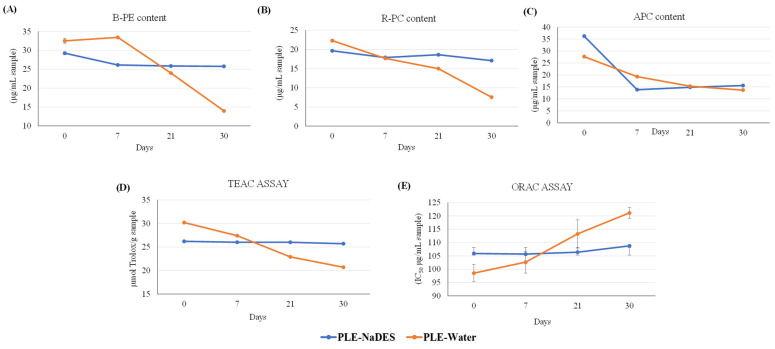
Forced stability study of (**A**) total B-phycoerythrin content, (**B**) total R-phycocyanin content, (**C**) total allophycocyanin content, (**D**) antioxidant capacity determined by TEAC, and (**E**) ORAC assays of PLE-NaDES and PLE-water extracts from step 1 of the biorefinery process submitted to 40 °C for 30 days.

As can be observed in Figure 3, the B-PE and R-PC values decrease up to three times in the PLE-water extracts from the first week to the last week compared with the PLE-NaDES extract (see Figure 3A,B).

However, the contents of these phycobiliproteins in the PLE-NaDES extracts were slightly reduced over time. By contrast, the low stability of APC was observed because its content was reduced from 36.2 ± 0.1 µg/mg sample to 15.60 ± 0.01 µg/mg sample in the PLE-NaDES extracts, with this reduction being more pronounced in the PLE-water extract (see Figure 3C). This means that the NaDES provided a protective effect on phycobiliproteins’ stability, particularly for B-PE and R-PC. This effect was also observed for the antioxidant capacity of hydrolysate extracts, because the TEAC and ORAC values were maintained over time in the PLE-NaDES extracts, while this capacity decreased in the PLE-water extracts (see Figure 3D and Figure 3E, respectively). In fact, even though initially the PLE-water extracts showed a higher antioxidant capacity as determined by the ORAC assay, this capacity was reduced up to 20% at the end of the stability study, presenting higher IC_50_ values than the PLE-NaDES extracts.

On the other hand, as can be seen in Table 4, the color measurements in the PLE-NaDES showed that L* and α* parameters were maintained during the stability study. These results indicated that the NaDES offers a protective effect on the degradation of phycobiliproteins over time, keeping its pink color, as can be observed in the measurement of the α* value. An increase in b* values is noted, indicating a more intense yellow color, particularly at 21 days.

By contrast, the color measurement of the PLE-water extracts varied significantly over time. The L* values increased from 760 to 968 and b* values from 61 to 278 in the PLE-water extracts, resulting in dark and blue extracts, respectively. Moreover, α* values decreased progressively, favoring the appearance of green colors. In fact, ΔE values indicated a slight color variation in the PLE-NaDES extracts of 56 ± 2, while for PLE-water, this variation was bigger (345 ± 3). This means that the NaDES exerts a protective effect against the degradation of compounds extracted by PLE that is not offered by conventional extraction solvents such as water.

## 3. Discussion

*P. palmata* is an underexplored red macroalga that is interesting for its potential and utilization as a source of phycobiliproteins, proteins, sulfated polysaccharides, and polyphenols. Usually, these components have been obtained from *P. palmata* using conventional extraction techniques with water as the extraction solvent [12,13,38]. The extraction solvent selection not only determines the efficiency of the extraction but also the integrity and stability of the compounds of interest. In fact, it is known that the color of phycobiliproteins is unstable with heat, acid, and light [19]. Thus, several authors have focused their efforts on the encapsulation of phycobiliproteins or on the addition of preservatives such as benzoic acid or citric acid, among others, to preserve their color integrity [39,40]. However, these are processes that significantly increase production costs.

In addition, advanced extraction techniques such as EAE have been applied to break down the cell wall of the algae to facilitate its destruction, increasing the extraction of proteins and phycobiliproteins [2]. Nevertheless, EAE can be a complex process, requiring the precise control of temperature, substrate concentration, and pH, which depend on specific enzymes that can be expensive and sensitive to degradation. For this reason, the use of the NaDES as the extraction solvent for the recovery of phycobiliproteins, proteins, and sulfated polysaccharides was proposed for this study. NaDESs are composed of natural compounds such as amino acids, sugars, or organic acids that are easy to obtain, cheap, and biodegradable [25]. In addition, NaDESs have shown an important stabilizing activity against the degradation of bioactive compounds, such as phenolic compounds [41]. However, the stabilizing properties of these solvents against phycobiliprotein degradation have not been studied yet. Taking into account the advantages of NaDES, its use could reduce the environmental impact of extraction processes compared to the use of industrially produced enzymes, the utilization of toxic volatile organic solvents, or other conventional solvents, without preservative properties against the degradation of target compounds. Moreover, the use of NaDES combined with advanced extraction techniques such as UAE or PLE could increase the breakage of algae cell walls by the ultrasound waves as well as by the high pressure applied during the extraction, respectively [42]. However, to our knowledge, this combination has not been applied for the recovery of phycobiliproteins, proteins, sulfated polysaccharides, and polyphenols from *P. palmata*. For these reasons, a biorefinery process was developed to obtain different fractions of high added-value compounds from *P. palmata* by using NaDESs combined with UAE and PLE.

### 3.1. Extraction of Phycobiliproteins, Proteins, and Sulfated Polysaccharides from P. palmata (Step 1)

The first step of the biorefinery process targeted the extraction of phycobiliproteins, proteins, and sulfated polysaccharides from *P. palmata*. One of the most important parameters that influence the recovery of phycobiliproteins is the extraction temperature [43]. High temperatures could improve solvent penetration into the matrix, increasing the extraction yield of target compounds. However, thermolabile compounds, such as phycobiliproteins, can be degraded at high temperatures. In fact, extraction temperatures below 50 °C are recommended to maintain the stability of the pigments [24]. For this reason, the first step of the biorefinery process was carried out at low extraction temperatures (25 °C) to prevent the pigments’ degradation.

In order to select the best extraction technique combined with NaDESs for the recovery of phycobiliproteins, proteins, and sulfated polysaccharides in the first step of the biorefinery process, different NaDESs were tested with UAE and PLE. Moreover, the different NaDESs were synthetized with 25% and 50% water, providing different physicochemical properties (viscosity, density, and polarity) using the same components. Nevertheless, the nature of NaDESs made PLE difficult; for instance, Gly:Glu:Bet and Gly:Glu:Pro NaDESs with only 25% water produced an obstruction during the extraction process. Even though these NaDESs provided lower viscosity values than the rest of the NaDESs (see Table S1), other physicochemical parameters such as the density or the nature of each NaDES component could have produced the obstruction of the PLE instrument. Thus, both NaDESs could not be implemented in step 1 of the biorefinery process using PLE. The results indicated that PLE using the Gly:Glu NaDES with 50% water provided extracts with the highest phycobiliprotein contents (see Table 1). In this sense, the application of a high pressure by PLE along with the physicochemical properties of the Gly:Glu NaDES with 50% water could have provided a better disruption of the cell structure of *P. palmata* compared to the ultrasonic waves of UAE, favoring the release of target compounds into the solvent [44]. Moreover, the addition of high percentages of water in the NaDES (50% instead of 25%) increased the mass transfer of target compounds by a reduction in NaDES viscosity and surface tension, improving the extraction of phycobiliproteins [35]. However, the PLE-water extraction provided extracts with higher protein and sulfated polysaccharide contents compared to the PLE-NaDES, UAE-NaDES, and UAE-water extracts. In fact, the hydrolysate of the PLE-water extracts presented the highest antioxidant capacity, as determined by the ORAC assay (see Table 2). This could be due to the fact that the PLE-water extraction allowed us to obtain a higher content of antioxidant water-soluble proteins. The solubility of proteins is strongly influenced by the molecular weight and the polar and nonpolar amino acids present in their structure [45]. Probably, the use of water submitted to a high pressure allowed us to break the cell wall of *P. palmata,* releasing higher polar protein and sulfated polysaccharide contents to the extraction solvent than with the use of NaDESs. Moreover, the high viscosity of the NaDES could have reduced the diffusion coefficients of proteins and sulfated polysaccharides, producing low mass transfer [27]. In fact, Table S1 shows the high viscosity of the NaDESs at room temperature, which is reduced when a temperature of 40 °C was applied. At moderately elevated temperatures, the solubility of proteins in NaDESs may improve by increasing the kinetic energy of molecules and by modifying the NaDES structure, facilitating its ability to solubilize proteins. Thus, higher extraction temperatures were considered for step 2 of the biorefinery process to maximize the recovery of non-thermolabile phycobiliproteins along with proteins and sulfated polysaccharides from the biomass residue of step 1.

### 3.2. Maximizing the Recovery of Phycobiliproteins, Proteins, and Sulfated Polysaccharides from P. palmata (Step 2)

Taking into account that the PLE-NaDES extractions provided the highest recovery rates of phycobiliproteins in step 1, UAE-NaDES extraction to maximize the recovery of phycobiliproteins, proteins, and sulfated polysaccharides from the residue of step 1 was not considered. In this sense, six different NaDESs were tested in combination with PLE for step 2 of the biorefinery process. Moreover, two PLE extraction temperatures were tested (25 °C and 40 °C) in order to determine their influence on the recovery of target compounds. The results indicated that the NaDES composed of Gly:Glu (2:1) with 50% water at 40 °C provided the extracts with the highest phycobiliprotein contents, as in step 1. This fact could be due to the Gly:Glu NaDES’s polarity, viscosity, density, pH, or its sugar composition, which could have improved its interaction with phycobiliproteins through hydrogen bonding and other interactions, improving its solubility and extraction. In fact, the extraction efficiency of this NaDES has been previously demonstrated by Hilali et al. [46], who evaluated the recovery of phycocyanin from *Arthrospira platensis* using UAE combining different glycerol-based NaDESs. Furthermore, the use of higher temperatures (40 °C) decreases the viscosity of NaDESs (Table S1), allowing for better penetration in the matrix and improving the extraction of target compounds.

On the other hand, comparing Table 1 and Table 3, it can be observed that the PLE-NaDES extraction in step 1 probably degraded the cell wall of *P. palmata,* facilitating the release of mainly sulfated polysaccharides, which can be recovered in step 2, as it is confirmed by the higher content of sulfated polysaccharides in the step 2 extracts (up to three times higher than in step 1). This effect was also observed for the extraction of proteins, but to a lower extent. In general, the use of PLE-NaDESs at 40 °C increased the extraction of proteins and sulfated polysaccharides compared with the use of PLE-NaDESs at 25 °C. This means that an increase in the extraction temperature facilitated the solubilization of proteins in the NaDESs [47]. Nevertheless, the PLE-water extracts presented higher total protein and sulfated polysaccharide contents than the PLE-NaDES extract, although with lower contents of phycobiliproteins. Thus, PLE-NaDES extraction using Gly:Glu (2:1) with 50% water was selected as the best extraction technique for the recovery of phycobiliproteins, proteins, and sulfated polysaccharides from *P. palmata* in step 1 and step 2 of the biorefinery process.

### 3.3. Recovery of Phenolic Compounds from the Residue of Phycobiliproteins, Protein, and Sulfated Polysaccharide Extractions

In general, the phenolic content of red algae is low (< 4% dry weight); however, *P. palmata* has demonstrated interesting contents in antioxidant phenolic compounds, although this has not been widely explored [48,49]. Particularly, ChCl:Gly (1:2) NaDES has been widely used for the extraction of phenolic compounds from natural sources [25,35,50]. This favorable behavior might be attributed to the strong interaction of hydrogen bonds between chemical groups of ChCl and Gly with phenolic compounds, increasing its extraction [51]. Nevertheless, its viscosity can reduce the penetration of the NaDES into the matrix, decreasing the extraction efficiency. For this reason, the water percentage in the NaDES is added to reduce its viscosity. In this sense, Domínguez-Rodríguez et al. [35] observed that the extraction of phenolic compounds increased with the addition of water in ChCl:Gly (1:2) NaDES and determined that 60% water in NaDES combined with PLE at 150 °C for 25 min provided extracts with the highest antioxidant and anticholinergic phenolic compound extracts from *Citrus reticulata* leaves. Thus, these extraction conditions were selected to extract the biomass residue obtained after step 2, targeting bioactive phenolic compounds.

Figure 2 shows that TPC values from the PLE-NaDES extract were within the range of the TPC values reported by other authors from a direct extraction of phenolic compounds from *P. palmata* (between 5 and 13 mg GAE/g extract) [52,53,54]. This means that the biorefinery process developed in this study allowed for the selective separation of different components from *P. palmata*, maximizing the use of biomass by extracting all valuable compounds. First, phycobiliproteins, proteins, and sulfated phycobiliproteins were obtained in step 1 and 2, preserving their degradation, followed by the extraction of phenolic compounds in step 3. As can be seen in Figure 2, TPC values obtained in PLE-NaDES extract were slightly higher than those reported in the literature data. Moreover, this study confirmed also that NaDES interacts with antioxidant phenolic compounds determined by a TEAC assay, increasing its extraction compared with ethanol/water (70:30, *v*/*v*) solvent, obtaining higher TPC values in PLE-NaDES than in ethanolic PLE extracts. However, the ethanolic extracts presented a higher antioxidant capacity determined by an ORAC assay as well as a higher anticholinergic capacity than the PLE-NaDES extract. This means that different phenolic compounds were obtained depending on the extraction solvent. The NaDES provided extracts that were more able to neutralize ABTS radicals, while ethanol/water (70:30, *v*/*v*) allowed us to obtain extracts with a higher capacity to neutralize O_2_ species. Probably, the polarity and low viscosity of the ethanol/water solvent promoted the extraction of more polar compounds different from phenolic compounds with a higher antioxidant capacity (as determined by an ORAC assay) and anticholinergic capacity than those obtained with the NaDES.

### 3.4. Preservative Effect of NaDES against Phycobiliprotein Degradation

Phycobiliprotein extracts and, in particular, the PLE-NaDES extract obtained using Gly:Glu (2:1) NaDES in step 1 were submitted to a forced stability study to determine the potential protective effect of NaDES on the degradation of these compounds; PLE-water extract exposed to the same conditions was employed as a control. The results indicated (see Figure 3) that the PLE-NaDES extract maintained the phycobiliprotein contents, the antioxidant capacity of its hydrolysate, and the color of the extract over time, while the PLE-water extract showed a reduction in all the tested parameters. This preservative effect of NaDES against the oxidative and thermal degradation of phycobiliproteins could be due to the high viscosity of this solvent compared with water [55,56]. An increase in the viscosity of NaDES produces a decrease in the mobility of molecules, reducing their exposure time to oxygen and avoiding their degradation [57]. In fact, the PLE-water extract presented a more intense yellow-brown color over time, which could indicate the oxidation of the extract by exposure to oxygen (see Table 4). This stabilizing effect can be adjusted by reducing the water content in the NaDES to increase the formation of hydrogen bonds between the target compounds and the NaDES [56]. However, the reduction in water content could affect the extraction efficiency of the target compounds. Thus, the water content in the NaDES is a crucial parameter to optimize in order to obtain stabilized extracts rich in target compounds.

## 4. Materials and Methods

### 4.1. Chemicals and Samples

Gallic acid, ascorbic acid, Trizma hydrochloride (Tris-HCl), trolox, disodium phosphate (Na_2_HPO_4_), monopotassium phosphate (KH_2_PO_4_), fluorescein sodium salt, sodium hydroxide (NaOH), sodium carbonate (Na_2_CO_3_), potassium persulfate (K_2_S_2_O_8_), acetylcholinesterase (AChE) Type VI-S from *Electrophorus electricus*, 2,2′-azino-bis(3-ethylbenzothiazoline-6-slphonic acid) (ABTS), toluidine blue, bovine serum albumin, and Bradford Reagent were supplied by Sigma-Aldrich (Madrid, Spain). Ethyl acetate (ETAC), ethanol (EtOH), methanol, and formic acid were purchased from VWR Chemicals (Barcelona, Spain). Folin–Ciocalteu reagent was acquired from Merck (Darmstadt, Germany) while acetylthiocholine iodide (ATCI), galantamine hydrobromide, 4-(amino-sulfonyl)-7-fluoro-2,1,3-benzoxadiazole (ABD-F), 2,2-azobis(2-aminodinopropane) dihydrochloride (AAPH), 2,2-difenil-1-picrilhidracilo (DPPH), choline chloride (ChCl), glucose, betaine, and proline were purchased from TCI Chemicals (Tokyo, Japan). Glycerol was provided by Labkem (Barcelona, Spain). Fucoidan (from Fucus serratus algae) was acquired from Carbosynth Limited, Berkshire, UK). Trypsin was purchased from MP Biomedical (Santa Ana, CA, USA).

The ultrapure water used (18.2 MΩ/cm) was obtained from a Millipore system (Millipore, Billerica, MA, USA).

Dried *Palmaria palmata* was provided by Porto Muiños (Cerceda, A Coruña, Spain). *P. palmata* powder was vacuum-packed and stored at −20 °C.

### 4.2. Proposed Biorefinery Process for the Valorization of P. palmata

In order to recover bioactive compounds from *P. palmata* with green solvents, two different approaches were used: UAE and PLE, both combined with green solvents such as water and NaDES. Figure 1 (in the Results section) illustrates a scheme of the proposed processes for the valorization of the algae.

A sequential process composed of three steps was designed for the extraction of phycobiliproteins, sulfated polysaccharides, and phenolic compounds from *P. palmata* as follows.

Step 1: The first step involved a comparative study between PLE and UAE to select the best extraction technique for the recovery of phycobiliproteins and sulfated polysaccharides from the alga. In this step, different NaDESs were tested and compared with water as extraction solvent. For this, a DIONEX ASE 200 (Dionex, Sunnyvale, CA, USA) was used for PLE extractions. PLE extractions were achieved in 11 mL extraction cells, which were filled with 1 g of *P. palmata* using 10.5 MPa for 20 min at 25 °C following Gallego et al. [24]’s method for the recovery of phycobiliproteins and sulfated polysaccharides from *Porphyridium cruentum* biomass. In order to compare PLE with UAE, 100 mg of *P. palmata* was mixed with 10 mL NaDES or water and sonicated at a frequency of 40 kHz at 25 °C for 50 min according to the optimized extraction methods of Domínguez-Rodríguez et al. [35] and Hilali et al. [46] for the extraction of bioactive compounds and phycobiliproteins from *Citrus reticulata* leaves and *Arthrospira platensis,* respectively.

Step 2: The residual biomass of the best extraction in terms of phycobiliprotein contents from step 1 was employed to extract the remaining phycobiliproteins and sulfated polysaccharides from *P. palmata*. In this step, the same NaDESs from the first step were tested and compared with water as extraction solvent. In addition, two temperatures were tested (25 °C and 40 °C) to obtain phycobiliproteins and sulfated polysaccharides.

Step 3: Phenolic compounds were recovered from the residual biomass of step 2 by PLE-NaDES using choline chloride:glycerol (ChCl:Gly) in a 1:2 molar ratio with 60% water in NaDES for 25 min at 150 °C and 10.5 MPa, according to Domínguez-Rodríguez [35]’s protocol. Additionally, to determine the efficiency of the use of NaDESs on the recovery of phenolic compounds from the residual biomass of step 2, the same PLE extraction conditions were applied but using ethanol:water (70:30, *v*/*v*) as solvent extraction instead of NaDES.

All extracts were centrifuged at 12,000× *g* for 15 min at 10 °C, the supernatant was removed, and they were stored at −20 °C until their use.

### 4.3. Preparation of Natural Deep Eutectic Solvents (NaDESs)

According to NaDESs employed in the literature data for the extraction of proteins and phycobiliproteins from algae, NaDESs presented in Table 5 were selected and formed following the protocol of Rajha et al. [58]. Each NaDES was synthesized using 25 and 50% water to obtain NaDESs with different viscosities. The mixture was heated for 1 h at 80 °C until a clear liquid was obtained.

### 4.4. Hydrolysis of Protein Fraction for In Vitro Assays

UAE-NaDES and PLE-NaDES extracts from step 1 were hydrolyzed using trypsin enzyme for obtaining bioactive peptides following the protocol described by Liu et al. [14]. Briefly, each 1 mL of extract was homogenized with 1 mL of phosphate buffer at pH of 8 for 10 min. Then, trypsin was added according to the total extract amount so that the enzyme/substrate ratio would be 4% (*w*/*w*). This mixture was incubated in a thermomixer (Eppendorf AG, Hamburg, Germany) at 37 °C to initiate the digestion process. The hydrolysate was recovered at 1, 3, 5, and 6 h of incubation in order to determine the optimal hydrolysis time. After incubation periods, hydrolysis was stopped by heating them at 95 °C for 10 min, then they were placed on ice, followed by cooling them to room temperature. Finally, the hydrolysates were centrifuged at 8000× *g* for 15 min, and the supernatants were removed and stored until their analysis.

### 4.5. Preparation of Phenolic Extracts for In Vitro Assays

To avoid NaDESs interfering with the analytical methods employed in this investigation for the study of phenolic compounds, phenolic extracts were purified by employing solid-phase extraction (SPE) according to Domínguez-Rodríguez et al. [25]’s method to remove NaDESs from the extracts. Briefly, C-18 cartridges (Strata C18-T, 500 mg) were placed in a vacuum system and equilibrated with 2.5 mL methanol (100%), followed by 7.5 mL of acidified water with formic acid (0.35%, *v*/*v*). After loading 2.5 mL of the sample, the cartridges were flushed with 2.5 mL of acidified water with formic acid (0.35%, *v*/*v*) to remove NaDESs. Then, phenolic compounds were eluted with 5.0 mL of ethyl acetate and 2.0 mL of acidified methanol with formic acid (0.1%, *v*/*v*). The phenolic fraction was dried with nitrogen and stored at −20 °C.

### 4.6. Spectrophotometric Analysis

#### 4.6.1. Determination of Total Protein Content

Bradford protein assay was employed to determine the total protein content of *P. palmata* extracts from steps 1 and 2 following the method described by Bradford [59]. Briefly, 5 μL of extract was mixed with 5 μL NaOH (1 M, pH: 8.0), and 250 μL of Bradford reagent was added. After 10 min in the dark at room temperature, absorbance was measured at 595 nm. Protein concentration was calculated by interpolation with a calibration curve carried out with bovine serum albumin (BSA) standard, and the results are expressed as mg protein/g sample.

#### 4.6.2. Determination of Phycobiliprotein Contents

Phycobiliprotein (B-Phycoerythrin (B-PE), Allophycocyanin (APC), and R-Phycocyanin (R-PC)) contents in the extracts of *P. palmata* from steps 1 and 2 were calculated using Roman et al. [60]’s method. The extracts were diluted at 1 mg/mL with water and the absorbance was measured at 620, 650, and 565 nm to determine the R-PC, APC, and B-PE contents, respectively. The results were expressed in mg phycobiliprotein/mL sample and calculated with the following equations:

R-phycocyanin [R-PC] = (Abs 620 nm − 0.7 × Abs 650 nm)/7.38(1)

Allophycocyanin: [APC] = (Abs 650 nm − 0.19 × Abs 620 nm)/5.6(2)

B-Phycoerythrin: [B-PE] = (Abs 565 nm − 2.8 × [R-PC] − 1.34 × [APC])/12.27(3)

#### 4.6.3. Determination of Sulfated Polysaccharides

The quantification of sulfated polysaccharides was carried out using the method described by Beutler et al. [61] and Albano et al. [62]. The extract (200 μL) was mixed with 1 mL of 0.01 mg/mL toluidine blue solution. After 10 min at room temperature, the absorbance of the mixture was measured at 620 nm. A calibration curve was utilized with fucoidan, and the results are expressed as mg sulfated polysaccharides/g sample.

#### 4.6.4. Determination of Total Phenolic Content

The total phenolic content in the phenolic extracts obtained by PLE-NaDES in step 3 was measured utilizing the Folin–Ciocalteu method, based on the procedure established by Singleton et al. [63]. A standard curve was generated using gallic acid in the range of 0.05–2 mg/mL to present the results as milligrams of gallic acid equivalents (GAE)/ sample.

### 4.7. Biological Activities

#### 4.7.1. Antioxidant Capacity

Reactive oxygen species (ROS) scavenging capacity according to ORAC assay: The antioxidant capacity of the extracts was determined by ORAC method described by Qu et al. [64]. Briefly, 100 μL of extracts at different concentrations was added to a mixture containing 100 μL of AAPH (590 mM) prepared in 30 mM phosphate buffer at a pH of 7.5 and fluorescein at a concentration of 10 μM in 25 μL phosphate buffer. The reaction was started at 37 °C, and fluorescence measurements were recorded every 5 min for 1 h (λexcitation = 485 nm; λemission = 530 nm). The peroxyl radical scavenging capacity of the extracts was calculated using the difference between the area under the curve (AUC) of fluorescence reduction and the percent inhibition obtained in the presence (AUCextract) or absence (AUCcontrol) of the extract according to the following equation:

(4) $$\%Inhibition=\frac{AUCcontrol-AUCsample}{AUCcontrol}\times 100$$

AUC was calculated by mean equation below:

(5) $$AUC=0.5+\sum f_{i}/f_{0}$$

where *f*_0_ and *f_i_* are fluorescence measurements at t = 0 min and every 5 min, respectively.

Trolox equivalent antioxidant capacity (TEAC) assay: TEAC assay was carried out using the method described by Re et al. [65]. ABTS stock solution was used, mixing 7 mM ABTS with 2.45 mM potassium persulfate. It was maintained under darkness for 12–16 h at room temperature. After that, the ABTS working solution was prepared by mixing 5 mM phosphate buffer (pH 7.4) with ABTS stock solution until absorbance at 734 nm reached values of 0.70 AU. Then, 10 µL of extract was added to 990 µL of ABTS working solution. Absorbance was measured at 734 nm after 45 min. A calibration curve was made employing Trolox as reference standard to express the results as TEAC (mmol Trolox/g sample). TEAC values were obtained from a calibration curve of each sample achieved from four different concentrations, which showed a liner response between 20 and 80% compared with the initial absorption.

#### 4.7.2. Anticholinergic Activity

To determine the inhibitory effect on acetylcholinesterase (AChE) activity, a fluorescent enzyme kinetics assay based on a modified version of Ellman et al. [66]’s method was applied [67]. The extracts were prepared at seven different concentrations ranging from 25 to 250 μg/mL by dissolving them in EtOH/H_2_O (1:1, *v*/*v*). A total of 100 μL of Tris-HCl buffer (150 mM, pH 8), 25 μL of AChE enzyme (0.8 U/mL), and 100 μL of extract were added to each reaction well, and the mixture was incubated at room temperature for 10 min. After incubation, 25 μL of ABD-F (125 μM) and 50 μL of acetylthiocholiniodide (ATCI) at the concentration calculated by Michaelis–Menten constant (KM) were added to each well. The reaction mixture was monitored by taking fluorescence measurements at 389 nm excitation and 513 nm emission wavelengths every minute using a microplate reader (Cytation 5, BioTek Instruments, Winooski, VT, USA) for 15 min at 37 °C. The percentage of enzyme inhibition was calculated using the mean velocities measured for AChE enzyme in the absence (V0) and presence (V1) of the sample according to the following equation:

(6) $$\%Inhibition=\frac{V0-V1}{V0}\times 100$$

The concentration of the extract that inhibited the enzyme activity by 50% (IC_50_) was determined in μg/mL, and it was observed that higher IC_50_ values indicated lower anticholinergic capacity. Galantamine hydrobromide at a concentration of 0.0125 mg/mL was used as a positive control. All measurements were performed in triplicate, and the results were reported as mean ± standard deviation (SD).

### 4.8. Stability Study

In order to observe the preservation effect of NaDESs on phycobiliproteins’ contents and the antioxidant capacity of their hydrolysates, a stability study following the protocol of Hilali et al. [46] was performed. The NaDES extract with the highest antioxidant phycobiliprotein content and water extracts were stored and submitted to accelerated aging conditions at 40 °C for 30 days, equivalent to 6 months at room temperature. Every 7 days, aliquots of extracts were collected and the phycobiliprotein concentrations (determined following the protocol described in Section 4.6.2), the antioxidant capacity of their hydrolysates (measured by TEAC and ORAC assays), and the color change of the extracts were monitored.

The color measurements were carried out during the stability test according to Idowu et al. [68]’s protocol using a Konica Minolta portable colorimeter (model CR-400), with D65 illuminant and an observation angle of 2°. Results were expressed according to CIELAB model, in which L* represents lightness (L* = 0 corresponds to the darkest, while L* = 100 is the brightest), α* represents the range color from red with positive values to green with negative values, and b* represents the color channel from the yellow positive values to the blue negative values. The Chroma Meter was initially calibrated in the CIELAB color space system employing a white tile. Three readings were taken for each sample. Color differences between the initial extract and the extract during the stability study were determined according to the following equation:

(7) $${{∆E}^{*}}_{Lab}=\sqrt{{\left(L2-L1\right)}^{2}+{\left(\alpha 2-\alpha 1\right)}^{2}+{\left(b2-b1\right)}^{2}}$$

where *L*1 represents the luminosity of the initial extract, *L*2 provides the lightness/darkness of the extract during each week of the stability study, *α* is the redness or greenness of the initial extract, and *α*2 the redness or greenness of the extract in each week of the stability study, while *b*1 is the yellowness/blueness of the initial extract and *b*2 is the yellowness/blueness of the extract in each week of the stability study.

### 4.9. Statistical Analysis

Statistical analysis was carried out employing Statgraphics Centurion version XVII software (Statistical Graphics Corp, The Plains, VA, USA) to compare the total phycobiliprotein, protein, and total sulfated polysaccharide contents as well as the antioxidant and anticholinergic capacities and color changes of the extracts. To determine statistically significant differences (*p* ≤ 0.05) between mean values for different extracts at a 95% confidence level, an analysis of variance (ANOVA) with Fisher’s exact test was utilized.

## 5. Conclusions

The potential of the pressurized NaDES extraction process was demonstrated for the sequential recovery of different fractions of valuable compounds from *P. palmata*. A pressurized NaDES composed of Gly:Glu (2:1) with 50% water improved the selectivity of thermolabile phycobiliprotein contents in the first step of the biorefinery process, compared to UAE-NaDES extraction. This pressurized NaDES allowed us to maximize the recovery of phycobiliproteins using 40 °C as the extraction temperature in comparison with 25 °C in the second step of the biorefinery process, as well as to obtain interesting protein and sulfated polysaccharide contents. The efficiency of PLE-NaDES extraction in the recovery of phycobiliproteins, proteins, and sulfated polysaccharides was corroborated by comparison with PLE-water extracts, with water being the extraction solvent usually employed for the extraction of these compounds. Moreover, a preservative effect of the NaDESs against oxidative damage and thermal exposure was confirmed with a forced stability study compared to water extracts. Finally, the extraction of the residual biomass of the second step was performed using a PLE-NaDES with ChCl:Gly (1:2) compared to PLE with ethanol:water (70:30, *v*/*v*); this corresponded to the third step of the biorefinery process and provided extracts enriched in antioxidant and anticholinergic phenolic compounds. In summary, the results obtained demonstrated the efficiency of pressurized NaDESs compared with the use of UAE-NaDESs and conventional solvents as a green alternative for the valorization of *P. palmata* biomass through a new biorefinery process.

## Figures and Tables

**Table 1:** Total phycobiliprotein contents (µg/mg sample, R-phycocyanin, allophycocyanin, and B-phycoerythrin), total protein content (µg/mg sample), and total sulfated polysaccharide contents (µg/mg sample) from extracts obtained by UAE-NaDES and PLE-NaDES at 25 °C during step 1.

STEP 1	R-Phycocyanin (R-PC) (µg/mg Sample)		Allophycocyanin (APC) (µg/mg Sample)		B-Phycoerythrin (B-PE) (µg/mg Sample)		Total Protein Content (µg/mg Sample)		Total Sulfated Polysaccharide Content (µg/mg Sample)	
Solvents	UAE	PLE	UAE	PLE	UAE	PLE	UAE	PLE	UAE	PLE
Gly:Glu, 50% H_2_O	11 ± 2 ^Aa^	12.4 ± 0.7 ^Ab^	14 ± 2 ^ABb^	18 ± 2 ^Aa^	17 ± 2 ^Aa^	18 ± 1 ^Aa^	173 ± 15 ^Aa^	126 ± 5 ^Cb^	54.3 ± 0.8 ^Aa^	50 ± 2 ^Bb^
Gly:Glu:Bet, 50% H_2_O	10.7 ± 0.6 ^Aa^	10.5 ± 0.1 ^Ba^	13 ± 2 ^Bb^	15.4 ± 0.9 ^ABa^	15.6 ± 0.9 ^ABa^	15.3 ± 0.5 ^ABa^	158 ± 9 ^Aa^	85 ± 9 ^Db^	53.4 ± 0.3 ^Aa^	44 ± 2 ^Cb^
Gly:Glu:Pro, 50% H_2_O	10.5 ± 0.4 ^ABa^	10.7 ± 0.5 ^Ba^	16 ± 2 ^ABa^	13 ± 1 ^Bb^	15.8 ± 0.4 ^ABa^	15.6 ± 0.6 ^ABa^	164 ± 6 ^Aa^	145 ± 14 ^Bb^	44 ± 2 ^Bb^	50 ± 2 ^Ba^
Gly:Glu, 25% H_2_O	8 ± 1 ^BCa^	8.9 ± 0.6 ^Ca^	17 ± 2 ^Ba^	9 ± 2 ^Cb^	13 ± 2 ^CDa^	12.8 ± 1.0 ^Ba^	94 ± 13 ^Bb^	131 ± 15 ^Ca^	37 ± 5 ^Ca^	30 ± 2 ^Db^
Gly:Glu:Bet, 25% H_2_O	6.8 ± 0.4 ^C^	⎯	14 ± 2 ^AB^	⎯	10.5 ± 0.9 ^D^	⎯	18 ± 1 ^D^	⎯	32 ± 4 ^C^	⎯
Gly:Glu:Pro, 25% H_2_O	8.6 ± 0.2 ^ABC^	⎯	17 ± 1 ^A^	⎯	14.1 ± 0.1 ^BC^	⎯	59 ± 8 ^C^	⎯	37 ± 4 ^C^	⎯
H_2_O	9.7 ± 0.4 ^ABa^	10.7 ± 0.5 ^Bb^	13.8 ± 0.6 ^ABa^	13 ± 1 ^Ba^	16.5 ± 0.2 ^ABa^	15.6 ± 0.6 ^ABa^	152 ± 7 ^Ab^	189 ± 7 ^Aa^	38.4 ± 0.2 ^BCa^	61.7 ± 0.7 ^Ab^

^A,B,C,D^ Letters indicate statistically significant differences in the same column (*p* ≤ 0.05). ^a,b^ Letters indicate statistically significant differences between extraction techniques (*p* ≤ 0.05).

**Table 2:** Antioxidant capacity of UAE-NaDES, UAE-water, PLE-NaDES, and PLE-water extracts and their respective hydrolysates determined by TEAC (µmol trolox/g sample) and ORAC (IC_50_, µg/mL sample) assays.

	TEAC (µmol trolox/g Sample)				ORAC (IC_50_ μg/mL Sample)			
	Extracts		Hydrolysate		Extracts		Hydrolysate	
Solvents	UAE	PLE	UAE	PLE	UAE	PLE	UAE	PLE
Gly:Glu, 50% H_2_O	14.81 ± 0.02 ^Bb^	25.64 ± 0.04 ^Ba^	20.90 ± 0.01 ^Bb^	35.60 ± 0.02 ^Ca^	107 ± 7 ^Ba^	111 ± 9 ^Bb^	29 ± 2 ^Bb^	27.8 ± 0.8 ^Ba^
Gly:Glu:Bet, 50% H_2_O	14.39 ± 0.03 ^Bb^	25.32 ± 0.03 ^Ba^	19.30 ± 0.01 ^Cb^	46 ± 1 ^Aa^	111 ± 4 ^Ca^	112 ± 8 ^Ca^	32 ± 2 ^Cb^	28 ± 3 ^Ba^
Gly:Glu:Pro, 50% H_2_O	13.28 ± 0.07 ^Cb^	13 ± 0.02 ^Ca^	18.10 ± 0.02 ^Db^	40.17 ± 0.02 ^Ba^	116 ± 5 ^Da^	116 ± 3 ^Da^	31 ± 1 ^Cb^	28 ± 1 ^Ba^
H_2_O	15.74 ± 0.05 ^Ab^	27.48 ± 0.03 ^Aa^	22.30 ± 0.01 ^Ab^	42.05 ± 0.02 ^Ba^	101 ± 11 ^Ab^	94 ± 3 ^Aa^	22 ± 2 ^Ab^	15.4 ± 0.2 ^Aa^

^A,B,C,D^ Letters indicate statistically significant differences in the same column (*p* ≤ 0.05). ^a,b^ Letters indicate statistically significant differences between extraction techniques (*p* ≤ 0.05).

**Table 3:** Total phycobiliprotein contents (µg/mg sample, R-phycocyanin, allophycocyanin, and B-phycoerythrin), total protein content (µg/mg sample), and total sulfated polysaccharide contents (µg/mg sample) from extracts obtained by PLE-NaDES at 25 °C and 40 °C during step 2.

STEP-2	R-Phycocyanin (R-PC) (µg/mg Sample)		Allophycocyanin (APC) (µg/mg Sample)		B-Phycoerythrin (B-PE) (µg/mg Sample)		Total Protein Content (µg/mg Sample)		Total Sulfated Polysaccharide Content (µg/mg Sample)	
Solvents	25 °C	40 °C	25 °C	40 °C	25 °C	40 °C	25 °C	40 °C	25 °C	40 °C
Gly:Glu, 50% H_2_O	20 ± 1 ^ABb^	28 ± 2 ^Aa^	36 ± 2 ^Ab^	52 ± 8 ^Aa^	29 ± 1 ^ABb^	43 ± 4 ^Aa^	218 ± 8 ^Db^	268 ± 14 ^Da^	123 ± 2 ^Cb^	131 ± 3 ^Ba^
Gly:Glu:Bet, 50% H_2_O	20.1 ± 0.7 ^ABa^	20 ± 3 ^BCa^	35.4 ± 0.6 ^Aa^	35 ± 4 ^Ba^	29 ± 1 ^ABb^	30 ± 4 ^Ba^	181 ± 10 ^Ea^	176 ± 3 ^Ea^	119 ± 3 ^Db^	124 ± 3 ^Ca^
Gly:Glu:Pro, 50% H_2_O	22 ± 2 ^Aa^	20.9 ± 0.4 ^Bb^	28 ± 3 ^Ba^	25.3 ± 0.9 ^Cb^	32 ± 3 ^Aa^	30.4 ± 0.5 ^Bb^	357 ± 5 ^Ba^	330 ± 7 ^Cb^	129.9 ± 0.4 ^Ba^	131 ± 5 ^Ba^
Gly:Glu, 25% H_2_O	18.1 ± 0.4 ^Ba^	17.1 ± 0.4 ^Cb^	18.0 ± 0.9 ^Ca^	15.6 ± 0.9 ^Db^	26.1 ± 0.5 ^Ba^	24.5 ± 0.5 ^Cb^	292 ± 7 ^Cb^	387 ± 3 ^Ba^	89 ± 4 ^Eb^	100 ± 1 ^Da^
H_2_O	22 ± 2 ^Aa^	20.9 ± 0.4 ^Bb^	28 ± 3 ^Ba^	25.3 ± 0.9 ^Cb^	32 ± 3 ^Aa^	30.4 ± 0.5 ^Bb^	431 ± 5 ^Aa^	410 ± 2 ^Ab^	146.0 ± 0.7 ^Aa^	141.9 ± 0.4 ^Aa^

^A,B,C,D^ Letters indicate statistically significant differences in the same column (*p* ≤ 0.05). ^a,b^ Letters indicate statistically significant differences between extraction temperatures (*p* ≤ 0.05).

**Table 4:** Color parameters (L* = luminosity; α* = redness/greenness; b* = yellowness/blueness) of PLE-NaDES and PLE-water extracts during the forced stability study.

	PLE-NaDES				PLE-Water			
Days	L*	α*	b*	ΔE	L*	α*	b*	ΔE
0	838 ± 1 ^C^	179 ± 1 ^B^	69 ± 2 ^C^	0	760 ± 1 ^C^	233 ± 1 ^A^	61 ± 2 ^D^	0
7	841 ± 1 ^C^	105 ± 0 ^D^	51 ± 0 ^D^	76 ± 0 ^Ab^	755 ± 0 ^D^	76 ± 1 ^B^	112± 1 ^C^	165.3 ± 1 ^Ba^
21	864 ± 1 ^A^	162 ± 1 ^C^	81 ± 1 ^B^	33 ± 1 ^Cb^	974 ± 0 ^A^	59 ± 1 ^C^	268 ± 1 ^B^	344 ± 1 ^Aa^
30	844 ± 1 ^B^	183 ± 0 ^Aa^	125 ± 0 ^A^	56 ± 2 ^Bb^	968 ± 0 ^B^	64 ± 0 ^D^	278 ± 2 ^A^	345 ± 3 ^Aa^

^A,B,C,D^ Letters indicate statistically significant differences in the same column (*p* ≤ 0.05). ^a,b^ Letters indicate statistically significant differences between extraction solvents (*p* ≤ 0.05).

**Table 5:** Hydrogen bond acceptors (HBAs) and hydrogen bond donors (HBDs) used in the NaDESs’ compositions.

Abbreviations	HBA	HBD	Molar Ratio	Percentage of Water in NaDES	Reference
Gly:Glu	Glycerol	Glucose	2:1	25 and 50%	[46]
Gly:Glu:Bet	Glycerol	Glucose and Betaine	4:1:1	25 and 50%	[46]
Gly:Glu:Pro	Glycerol	Glucose and Proline	4:1:1	25 and 50%	[46]
ChCl:Gly	Choline chloride	Glycerol	1:2	60%	[35]

## Data Availability

The original contributions presented in this study are included in the article/Supplementary Materials; further inquiries can be directed to the corresponding authors.

## Supplementary Materials

The following supporting information can be downloaded at: https://www.mdpi.com/article/10.3390/md22100467/s1, Table S1: Viscosity values (mPas·s) of NaDES employed in this study with 25% and 50% of water measured at 25 °C and 40 °C. Table S2: Antioxidant capacity of PLE-Water and PLE-NaDES (Gly:Glu, 50% H_2_O) extract according to hydrolysis time determined by ORAC (IC_50_, µg/mL sample) analyses and TEAC (µmol trolox/g sample).
