# Supplementary material for: New Green Biorefinery Strategies to Valorize Bioactive Fractions from Palmaria palmata
Source: Mar Drugs. 2024 Oct 11;22(10):467. doi: 10.3390/md22100467 (PMC11509369; doi:10.3390/md22100467)
Supplement: Supplementary file 1 [file marinedrugs-22-00467-s001.zip › marinedrugs-3232871-supplementary.pdf]

New Green Biorefinery Strategies to Valorize Bioactive Fractions from *Palmaria palmata*

Melis Cokdinleyen [1,2], Gloria Domínguez-Rodríguez [1,3,*], Huseyin Kara [2], Elena Ibáñez [1] and Alejandro Cifuentes [1]

[1] Laboratory of Foodomics, Institute of Food Science Research (CIAL, CSIC), Nicolás Cabrera 9,
28049 Madrid, Spain; melis.cokdinleyen@cial.uam-csic.es (M.C.); elena.ibanez@csic.es (E.I.); a.cifuentes@csic.es (A.C.)
[2] Faculty of Sciences, Department of Chemistry, Selçuk University, Arciçh, Ismetpasa Cad, Selçuklu,
Konya 42250, Turkey; hkara@selcuk.edu.tr
[3] Departamento de Química Analítica, Química Física e Ingeniería Química, Facultad de Ciencias,
Universidad de Alcalá, Ctra. Madrid-Barcelona Km. 33.600, 28871 Alcalá de Henares, Madrid, Spain
* Correspondence: gloria.dominguezr@uah.es

Supporting Information (SI)

Table S1. Viscosity values (mPas·s) of NaDES employed in this study with 25% and 50% water measured at 25°C and 40°C.

	25°C (mPa·s)		40°C (mPa·s)	
**NaDES**	**25% H_2O**	**50% H_2O**	**25% H_2O**	**50% H_2O**
Gly:Glu	$34 \pm 2_{A}^{a}$	$11 \pm 1_{A}^{b}$	$16.1 \pm 0.1_{A}^{a}$	$6.6 \pm 0.3_{A}^{b}$
Gly:Glu:Bet	$31 \pm 1_{A}^{a}$	$10.0 \pm 0.7_{A}^{b}$	$20 \pm 1_{A}^{a}$	$7.8 \pm 0.3_{A}^{b}$
Gly:Glu:Pro	$30 \pm 3_{A}^{a}$	$7.9 \pm 0.3_{A}^{b}$	$17.9 \pm 0.3_{A}^{a}$	$6.6 \pm 0.3_{A}^{b}$

^{A,B,C,D} *Letters indicate statistically significant differences in the same column ($p \leq 0.05$).* ^{a,b,c,d} *Letters indicate statistically significant differences extraction solvents ($p \leq 0.05$).*

Table S2. Antioxidant capacity of PLE-Water and PLE-NaDES (Gly:Glu, 50% H_2O) extract according to hydrolysis time determined by ORAC (IC_{50}, µg/mL sample) analyses and TEAC (µmol trolox/g sample).

Hydrolysis Time	ORAC (IC_{50} µg/mL sample)		TEAC (mmol/g sample)	
	**PLE-NaDES**	**PLE-Water**	**PLE-NaDES**	**PLE-Water**
**1h**	$28 \pm 1_{A}^{b}$	$15.4 \pm 0.2_{A}^{a}$	$45.15 \pm 0.01_{A}^{a}$	$43.54 \pm 0.02_{A}^{b}$
**3h**	$30 \pm 2_{B}^{b}$	$21 \pm 3_{B}^{a}$	$43.10 \pm 0.02_{B}^{a}$	$40.05 \pm 0.03_{B}^{b}$
**5h**	$29 \pm 1_{B}^{b}$	$21 \pm 2_{B}^{a}$	$43.50 \pm 0.01_{B}^{a}$	$39.88 \pm 0.07_{B}^{b}$
**6h**	$30.1 \pm 0.5_{B}^{b}$	$24 \pm 3_{C}^{a}$	$42.35 \pm 0.01_{C}^{a}$	$40.50 \pm 0.01_{B}^{b}$

^{A,B,C,D} *Letters indicate statistically significant differences in the same column ($p \leq 0.05$).* ^{a,b,c,d} *Letters indicate statistically significant differences extraction solvents ($p \leq 0.05$).*
